# It’s what’s on the inside that counts: Capsid content as a critical quality attribute for AAV characterization

**DOI:** 10.1016/j.omta.2026.201765

**Published:** 2026-06-18

**Authors:** Lauriel F. Earley, Stuart Nelson, Ana Dolinar Češarek, David Dobnik

**Affiliations:** 1 Earley Stage Gene Therapy Consulting, Tacoma, WA, USA; 2 Eli Lilly & Co., Lilly Technology Center, Indianapolis, IN, USA; 3 National Institute of Biology, Ljubljana, Slovenia; 4 Niba Labs d.o.o., Ljubljana, Slovenia

## Abstract

Recombinant adeno-associated virus (rAAV) vectors exhibit complex heterogeneity in capsid content. Although a growing array of analytical platforms can interrogate different capsid attributes, no single method provides a comprehensive or unequivocal assessment. Critically, current approaches often fail to evaluate genome integrity, an emerging important quality attribute. Incomplete genomes can contribute to viral genome titers without conferring therapeutic benefit, while simultaneously increasing immunogenic burden and risk of adverse events. Discordant results from orthogonal assays further complicate interpretation and cross-laboratory comparisons. Here, we highlight the limitations of existing characterization strategies and advocate for robust analytical frameworks that directly assess genome completeness. We propose best-practice guidelines, including improved genome integrity assays and harmonized reporting, to better link capsid content analytics with potency and patient safety in gene therapy development.

## Introduction

Recombinant adeno-associated virus (rAAV) vectors, widely used in gene therapy, exhibit complex heterogeneity, including full capsids (containing a complete genome), empty capsids (lacking an encapsidated genome), partially filled capsids (containing fragments of genomes), and packaged genetic impurities derived from the host cell, helper DNA, or plasmid backbones.^1^^,^^2^ Next-generation sequencing (NGS) analyses reveal even greater heterogeneity, with a spectrum of poorly defined subpopulations.^3^ Although analytical methods continue to advance, no individual assay offers comprehensive or unequivocal assessment of capsid content. This article highlights the limitations of current approaches and calls for standardized, multi-attribute analytics targeting genome integrity to ensure product quality and patient safety.

A variety of platforms (such as analytical ultracentrifugation [AUC]), charge detection mass spectrometry (CDMS), anion exchange chromatography, cryo transmission electron microscopy (EM), mass photometry, next-generation sequencing (NGS), and third-generation sequencing (TGS), are used to characterize the content of AAV capsids. Each method interrogates different product attributes (mass, density, charge, and sequence) and has unique blind spots. For example, physical methods may distinguish capsids by mass or density but cannot identify the sequence or integrity of encapsidated genomes. NGS and TGS can evaluate sequence accuracy and relative quantitative data on genome types but can also introduce sequencing artifacts or miss some genomic subtypes.

**Figure 1:**
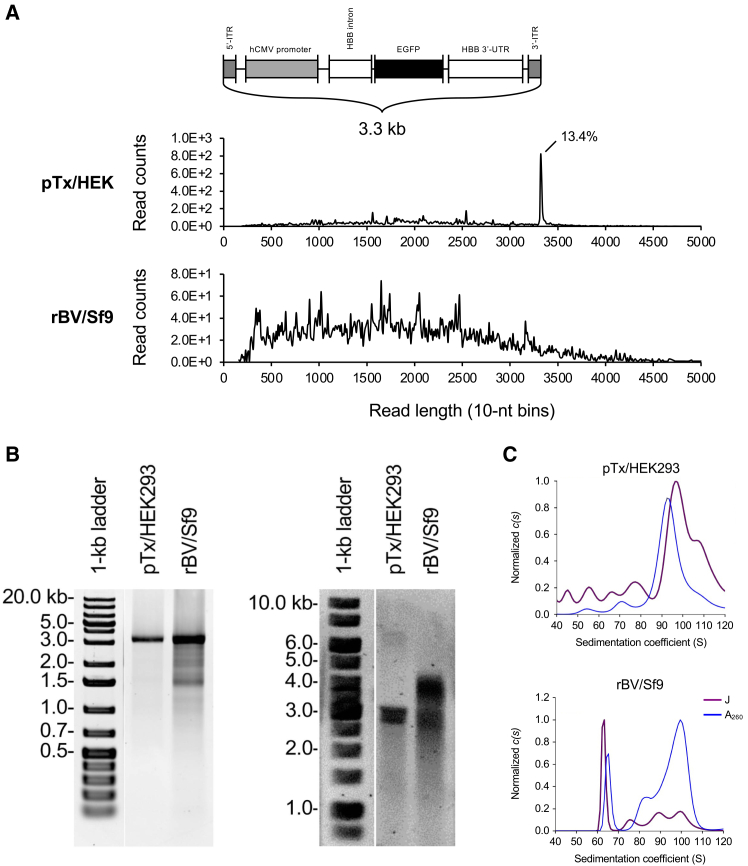
Example of orthogonal assay results for the same sample (pTx/HEK293 and rBV/Sf9) (A) Long-read sequencing results showing the frequencies of read-lengths in base pairs (bp) binned by 10 nt. For pTx/HEK293 sample, one prominent peak is visible at expected vector genome length (3300 bp) which corresponds to only 13% of all successfully mapped reads. Sample rBV/Sf9 has no prominent peak visible. (B) Native and alkaline gel electrophoresis images, showing predominantly one band at expected genome length and a smear of fragmented DNA. (C) AUC analysis identifying the majority of pTx/HEK293 sample particles as full (S around 100), some partial (S between 70 and 90) and empty (S around 65). The width of the full peak is relatively wide and could probably include a variety of different genome lengths. Sample rBV/Sf9 has a more pronounced empty fraction. Blue and purple signals respectively correspond to the sedimentation profile obtained by absorbance at 260 nm (A260) and interference fringe shift (J). Adapted from Tran et al.,^3^ with permission.

Therefore, until better multi-attribute analytical methods exist, a comprehensive product characterization strategy must rely on multiple orthogonal assays, the results of which will not always align since each assay measures different product attributes. One such example is shown in Figure 1, which serves to showcase analytical comparability of genome integrity results obtained by different methods. Differences in program design, capsid composition/origin, gene payload and length, manufacturing cell lines, and purification strategies can all influence the analytical profile of a given payload. To integrate these data into a clear picture, each assay should be well characterized, validated, and include proper controls. Unfortunately, this can be a daunting task as available reference material may not be applicable and more data points can often lead to greater uncertainty about which results are the most “true”.

Different instruments and the absence of harmonized standards means that two labs characterizing the same AAV batch can report very different results (e.g., titer or full:empty ratios).^1^^,^^4^^,^^5^^,^^6^ This limits the gene therapy field’s ability for cross-comparison. Doing so may misrepresent a true composition of vector preparations and their impact on clinical outcomes.

## Use of orthogonal methods is encouraged but may still mislead

It is often recommended to apply orthogonal analytical methods,^7^ but in practice, results from orthogonal platforms frequently diverge because each is sensitive to different attributes of the vector or may quantify slightly different species.

• Given the heterogeneous nature of capsid populations, categorization becomes challenging when analytics change. One method may classify a borderline particle as full, while another may call it partial due to differences in detection thresholds or resolution.
• Methods may vary in what they count as a genome-containing particle: PCR targets a specific primer set region, whereas AUC detects a mass difference. This means a partially filled capsid with a large fragment could register as full in AUC (due to density) but might be flagged as empty in PCR if the fragment lacks the targeted gene region, or vice versa.
• Different platforms also report different units (genome copies, optical density, percent full by area, etc.), each with unique calibration standards and quantitation approaches. Without a common reference, cross-comparison can be error prone.^1^^,^^8^
• Common sequencing methods can only measure blunt, double-stranded DNA, which may miss genomes that remain single-stranded at the library preparation stage. These genomes would still be measurable by other methodologies such as AUC or CDMS, but each of these assays will have very different data outputs.

Ultimately, none of the current assays are calibrated to 100% accuracy. Although necessary, until more comprehensive analytics exist, comparing them can be misleading. This has prompted calls for better standardization with the critical need for standardized reference materials and cross-platform calibration.^1^^,^^4^

## The problem of standardization

Results from orthogonal analyses must be interpreted with caution and even then, inter-laboratory testing of the same material can still yield different outcomes.^5^^,^^6^ On the industry side, organizations such as the National Institute of Standards and Technology (NIST) and National Institute for Innovation in Manufacturing Biopharmaceuticals (NIIMBL) have convened working groups to harmonize measurements.^8^ The recent interlaboratory study by Lehman et al. (2024) took a step in this direction: by having many laboratories measure identical, blinded samples using various techniques, it highlighted which methods are inherently more reproducible.^8^ This kind of data can guide where to focus standardization efforts, such as developing a uniform qPCR protocol and reagent set, possibly under an ISO or pharmacopeial guideline.

An additional complication is the availability and use of common reference standards, which are not applicable for most assays, as many are affected by and evaluate the transgene of interest. Regardless, we encourage the use of standards when applicable and available, especially for calibrating instruments or validating protocols. Currently, there are two AAV reference standards available through ATCC, which have been characterized for capsid titer, vg titer, and infectious units.^5^^,^^6^ However, the assays are limited to the AAV2 or AAV8 capsid and the pTR-UF11 vector genome sequence. Given that, these reference standard materials are still the most well-characterized available and could be enhanced with further assays for genomic integrity. In addition, a recent effort has been made towards creating a protocol for the generation of an AAV genomic standard.^9^

Finally, the field should align on terminology and reporting units. Terms like “partial” or “intermediate” capsids need clear definitions regarding therapeutic impact. For example, genomes arising from nicked self-complementary AAV could still provide the intended payload cassette but genomes missing a promoter will not. Yet, both of these very different genomic species are currently called “partial”, “intermediate”, or “truncated”. In another example, even “full” can have various meanings since the complete cassette can present as intended or as an “overfull” genome that contains the intended genome and extra genomic material such as partial concatenated genomes or plasmid backbones. In addition to payload terminology, reporting a full:empty ratio and how it was calculated should be standard practice. Even something as simple as agreeing to report the full capsid percentage (FTT, full-to-total) instead of full:empty, as Lehman et al. suggest,^8^ would reduce confusion (FTT provides a 0%–100% scale, whereas a ratio can be misinterpreted). Currently there is a working group within ISO developing a standard on characterization of capsid content that is set to clarify definitions and provide information on available methods and approaches. Additionally, as the field is developing more reference standards, as is currently being pursued by a joint effort between NIST, NIMBLE, and USP, we recommend that genomic integrity should be an included characterization assay for this material. The production and distribution of biological reference standards is currently limited, but in efforts to expand to a global hub, professional organizations like ASGCT and the AAV Reference Standard Working Groups could possibly coordinate with organizations like the National Institute for Biological Standards and Control to provide these much needed resources.

## Genome integrity and potency: The missing link

Perhaps the most critical relationship to understand is between vector genome integrity and biological potency. Manufacturers often quantify AAV dose as *viral genomes per milliliter* (VG/mL), usually measured by targeting a small part of the vector genome. This does not always correlate with the number of complete genomes, as many capsids carry incomplete genomes that contribute to the VG count without delivering therapeutic effect.^10^

Different types of fragmented genomes might have varying effects. Some may include regulatory elements but lack coding regions; others may be random host or plasmid DNA.^10^ These aberrant genomes could act as decoys or competitors, potentially competing with functional vectors for cellular machinery.^11^ One study even proposed a beneficial role for specific fragments, which could act as primers for second-strand synthesis in the transduced cell.^12^ Other fragments may simply be benign passengers. It remains largely unknown how each subgenomic species influences overall transgene expression, dose requirements, or immunogenicity. These are likely product dependent and could differ based on production processes, capsid serotype, promoter used, and gene of interest. A recent study showed that intermediate capsids (carrying incomplete genomes) are as infectious as full capsids *in vitro* but do not produce significant transgene expression or therapeutic effect.^10^ Therefore, genomic integrity can be considered a potency-related critical quality attribute.

This gap has direct clinical relevance. Dosing for gene therapy is a careful balance between efficacy and safety; if a significant fraction of administered VGs are non-functional, one might inadvertently overdose patients with excess capsid proteins to compensate, raising the risk of immune reactions.^10^ Indeed, empty capsids (which contain small fragments or no genome at all) are known to increase antigen load and have been correlated with heightened immune responses and adverse events in trials.^10^ It stands to reason that fragmented genome-containing particles could pose similar risks without benefit.^7^^,^^10^

Thus, understanding genome integrity is a key to truly linking product analytics with potency. Regulatory agencies, including the FDA, recognize both the sequence and length of the vector genome as typical potency-related critical quality attributes, underscoring the need for analytical methods that can accurately assess these parameters.^13^^,^^14^ We need assays that do not just count genomes but assess their length and completeness. Some emerging approaches aim to address this: for instance, long-read sequencing or multiplex digital PCR methods can map the internal genome fragments across a vector batch.^4^ By probe-hybridization one can compare coverage across the transgene and estimate what fraction of genomes are complete versus truncated at various points.^15^ Such methods, especially if coupled with reference standards or spike-in controls,^4^ could quantitatively profile genome integrity in a lot. While not yet routine, these tools could fill the potency knowledge gap by correlating specific fragment profiles with transduction outcomes.

Several groups, including consortia and contract development and manufacturing organizations are developing common reference vectors and protocols to harmonize the testing of different critical quality attributes.^4^^,^^8^ Currently, reference standards primarily address titers and full:empty ratios but not genome integrity.^9^ Moving forward, it would be beneficial to have reference AAV preparations enriched in partial genomes of known types (e.g., a batch where 50% of particles contain a defined 2 kb truncation). These could serve as controls to validate that new integrity assays like PCR panels or sequencing approaches are working as intended. Creating such standards is non-trivial,^9^ but collaborations between academia, industry, and standards bodies like USP^8^ can make it feasible.

## Recommended best-practices for capsid content analysis

To address these challenges, we propose the following framework for more reliable assessment of AAV capsid content as a best-practice guideline.

1. Employ analytical methodologies early in development that have QC potential later. Maintaining consistent analytical approaches across dose-ranging investigational new drug (IND)-enabling studies, non-clinical toxicology assessments, and clinical lot characterization, ensures standardized dosing metrics and capsid characterization throughout the product life cycle.
2. Incorporate genome integrity assays into routine characterization. This could be as simple as an alkaline gel on extracted AAV DNA or, preferably, a more product-specific assay such as multiplex digital PCR. Such assays are relatively straightforward and can be highly informative—a high titer comprised largely of incomplete genomes, such as those missing the 3′ end or any other vital part of the genome, would indicate a potency issue. We recommend developing standardized multi-site digital PCR panels for AAV genome integrity as a release or characterization test. Alternatively, other robust multi-target assays can be used.^15^
3. When needed, use multiple orthogonal methods in parallel to ensure the necessary attributes for completeness and sequence accuracy are evaluated. The goal is not to multiply assays for routine release but to understand your product as it progresses through development. When two complementary assays with different analytical approaches are employed to characterize capsid content, confidence in the measurement increases if results converge. If results diverge, do not average them; instead, investigate the cause. Carefully interpret orthogonal data: when multiple methods are used, discrepancies should be leveraged to glean insight. For instance, if AUC shows 20% partial but sequencing reveals a broad range of fragments, one might deduce that the partial category includes various non-functional genomes, which could impact potency. In all cases, err on the side of caution—if any method suggests a lower functional full capsid content, consider that as a possible true worst case in risk assessments. Divergent outcomes may indicate atypical genome forms or biases in what is being quantified; these should be reported and examined rather than glossed over. These methods should be appropriate for the products’ development phase and can be adjusted or expanded as development progresses.

By following these practices, developers can build a more complete and reliable understanding of their vector products and monitor them throughout the program lifespan. Structural elucidation, including transgene genome sequencing, should be documented at IND submission and updated as manufacturing processes evolve.

## Conclusions

Capsid content and genome integrity must be recognized as a key critical quality attributes that must be monitored throughout the drug product life cycle. Critically, the aim is not to increase testing burden but to deploy better, more informative methods that can replace or refine existing characterization approaches. To achieve this, assays should be well-controlled, thoroughly understood, validated, and capable of detecting any significant changes. The same rigorous and robust assays used for IND-enabling and toxicology studies should be applied to clinical material to avoid dosing patients with suboptimal material.

We strongly recommend that developers provide comprehensive capsid content and genome integrity data in product characterization dossiers. Rather than reporting a single “genome titer” value, data should be presented to show the composition of that titer. Accumulating such data over time will enable the field to correlate these features with clinical efficacy or toxicity. In summary, without improved analytics and understanding of genome fragment effects, capsid content metrics may not accurately reflect potency and could impact patient safety. The field should invest in the development and adoption of standardized reference materials, improved QC-friendly, multi-attribute assays, and collaborative studies to connect analytical data with *in vivo* effects. By doing so, we would ensure that “vector genomes per dose” truly reflect therapeutic benefit. We must end reliance on incomplete data and prioritize genome integrity for future gene therapies.

## Acknowledgments

The work leading to this manuscript was partially funded by the Slovenian Research and Innovation Agency (research core funding P4-0463 and project L4-3180).

## Author contributions

D.D. conceptualized the idea and prepared an initial outline of the manuscript. All authors contributed to discussions on the content and to writing of the text until its final version. A.D.C. prepared and obtained permission to publish content presented in Figure 1. All authors have revised the manuscript and approved the final version.

## Declaration of interests

D.D. acts as CSO for NibaLabs d.o.o., which provides analytical services and dPCR assays in the field of gene therapy. L.F.E. is an independent consultant at Earley Stage Gene Therapy Consulting. S.N. is an employee of Eli Lilly and Company.

## Declaration of generative AI and AI-assisted technologies in the writing process

During the preparation of this work the authors used Claude in order to propose a draft structure of the manuscript based on the prepared ideas and outline. After using this tool/service, the authors reviewed and edited the content as needed and take full responsibility for the content of the published article.
